# Left Cerebellar‐Right Frontal Uncoupling in Female First‐Episode Major Depressive Disorder: Synaptic Evidence From [ ^18^F]UCB‐H PET

**DOI:** 10.1002/cns.71144

**Published:** 2026-09-04

**Authors:** Juehan Wang, Yajun Lin, Tianfang Zhang, Pengfeng Xu, Shanyan Lei, Hong Yang, Zuobing Chen

**Affiliations:** ^1^ Department of Physical Medicine and Rehabilitation, The First Affiliated Hospital Zhejiang University School of Medicine Hangzhou China; ^2^ Department of Pain Management and Rehabilitation Zhejiang Cancer Hospital Hangzhou Zhejiang China; ^3^ Department of Radiology, Shanghai Children's Hospital, School of Medicine Shanghai Jiao Tong University Shanghai China; ^4^ Zhejiang Key Laboratory of Intelligent Rehabilitation and Translational Neuroelectronics Hangzhou China

**Keywords:** [^18^F] UCB‐H, cerebellum, major depressive disorder, network, PET

## Abstract

**Background:**

Synaptic loss is a neuropathological feature of depression.

**Objective:**

Cerebello‐cerebral synaptic function was evaluated in female first‐episode major depressive disorder (MDD) patients using [^18^F]UCB‐H positron emission tomography (PET).

**Materials and Methods:**

PET was performed on female first‐episode MDD patients (FMD group, *n* = 7), healthy controls (HC group, *n* = 9), and chronic restraint stress (CRS) mice (*n* = 9). Standardized uptake value ratios (SUVR), voxel‐wise tracer uptake analysis, functional connectivity (FC), and network analysis were performed at the cerebello‐cerebral level. Associations with Hamilton depression scale (HAMD) and Hamilton anxiety scale (HAMA) scores were assessed by Pearson correlation and logistic regression analysis.

**Results:**

In the FMD group, SUVR in the left cerebellum 7b was significantly reduced and negatively correlated with HAMD (*r* = −0.57) and HAMA (*r* = −0.55) scores. The cluster centered on the left cerebellar lobule VIII showed reduced tracer uptake and was negatively correlated with HAMD scores. FC was significantly reduced in the right frontal lobe but enhanced in the midbrain and occipital regions. The network was characterized by bilateral frontal lobe concentration in the FMD group, while the cerebellar network was more uniform. Cerebello‐cerebral uncoupling between the left cerebellum and the right frontal lobe was observed, manifested as reduced SUVR correlation and weakened FC. CRS mice also showed reduced brain [^18^F]UCB‐H uptake.

**Conclusions:**

Left cerebellar‐right frontal uncoupling may serve as a potential neural correlate of first‐episode MDD in females.

## Graphical abstract

This study investigates cerebello‐cerebral synaptic function in female first‐episode major depressive disorder (FMD) using [^18^F]UCB‐H PET imaging. Compared to healthy controls (HCs), FMD patients show reduced SUVR in the left cerebellar lobule VII and synaptic tracer uptake in the left cerebellar lobule VIII, which correlates negatively with depression and anxiety severity. Functional connectivity is decreased obviously in right frontal lobe. Network analysis reveals a more uniform cerebellar network in FMD. Critically, cerebello‐cerebral uncoupling, specifically between the left cerebellum and right frontal lobe, is identified as a key feature. These results suggest that disrupted left cerebellar‐right frontal circuitry may represent a neural signature of first‐episode MDD in females.

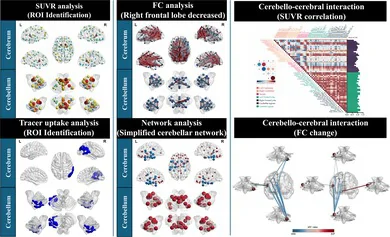

## 1 Introduction

The prevalence of depression among adolescents and young adults has risen steadily, with a reported lifetime prevalence of 20.6% [1]. Over the past three decades, depression cases have surged by 21.67%, with incidence rates increasing by 19.05% [2]. Major depressive disorder (MDD) affects approximately 246 million individuals worldwide [3, 4]. The high incidence and recurrence rates will likely impose substantial economic and healthcare burdens [5, 6]. Women are twice as likely as men to be diagnosed with MDD and have more severe symptoms [7]. Therefore, early detection and screening strategies for MDD, as well as investigation of its neuropathological mechanisms, are needed specifically in female cohorts.

Positron emission tomography (PET) imaging offers unique advantages for quantifying distinct molecular elements in the living brain, including receptors, transporters, and enzymes. Synaptic vesicle glycoprotein 2A (SV2A), a protein localized to synaptic vesicle membranes, has been established as a histological marker of synaptic density [8] and a validated PET biomarker for quantifying synaptic density [9]. Holmes et al. found that MDD patients had lower SV2A density than healthy controls (HCs), providing direct imaging evidence of impaired synaptic plasticity [10]. Furthermore, enhancing synaptic connectivity and plasticity has emerged as a promising therapeutic target for depression [11]. However, no definitive human PET studies have characterized SV2A levels in depression, and our understanding of the neuropathological mechanisms underlying SV2A synaptic alterations in MDD remains limited.

Previous neuroimaging studies have predominantly focused on supratentorial brain structures. However, advances in neuroimaging technology have substantially improved our understanding of the cerebellum's role in emotional function. Accumulating evidence indicates that the cerebellum participates in the pathophysiology of MDD, with pathological changes affecting multiple cerebellar subregions involved in cortical network communication and cognitive and self‐referential processing [12]. Cerebellar synaptic plasticity provides the structural foundation for functional connectivity (FC) between the sensorimotor, cognitive, and affective regions in the cerebellum and cerebrum [13]. Fang et al. first investigated the relationship between SV2A synaptic density and resting‐state networks in HCs [14]. Subsequently, only Lien et al. conducted preliminary research in old patients with depression, reporting increased betweenness centrality in the vermis 7 (Verm7) and decreased nodal degree in right cerebellum 4_5 (Cere4_5.R), reflecting reconfiguration of the emotional processing network. They also found that increased cerebellar FC may compensate for decreased prefrontal FC [15]. However, current understanding of SV2A changes in the cerebellum of young and middle‐aged MDD patients, as well as the mechanisms underlying cerebello‐cerebral interaction, remains extremely limited. Therefore, this study focused on female, first‐episode, MDD patients and employed [^18^F]UCB‐H PET imaging targeting SV2A to systematically investigate neuropathological changes in cerebello‐cerebral standardized uptake value ratios (SUVR), voxel‐wise analysis of tracer uptake, FC and network analysis.

## 2 Materials and Methods

### 2.1 Participants

Seven female patients with first‐episode MDD were enrolled in the FMD group. Diagnosis was confirmed independently by two licensed psychiatrists. Inclusion criteria were: (1) written informed consent; (2) age 18–75 years; (3) right‐handedness and native Chinese language proficiency; (4) meeting International Classification of Diseases, 10th Revision (ICD‐10) and Diagnostic and Statistical Manual of Mental Disorders, Fifth Edition (DSM‐5) criteria for depression; (5) first‐episode onset with no prior antidepressant treatment; (6) Hamilton depression scale (HAMD)–17 score ≥ 18 [16]. Nine volunteers matched for age, sex, and cultural background were recruited for the HC group. The non‐patient version of the Structured Clinical Interview for DSM‐5 was used to exclude HCs with a history of psychiatric or neurological disorders.

Exclusion criteria for all participants were: (1) history of epilepsy, substance abuse, or neuropsychiatric disorders; (2) pregnancy or menstruation at the time of scanning; (3) history of severe organic brain disease or intellectual disability; (4) MRI contraindications, including ferromagnetic implants or claustrophobia; (5) other neurological disorders, such as dementia or Parkinson's disease.

### 2.2 Neuropsychological Assessment

The HAMD [17] and Hamilton anxiety scale (HAMA [18]) were administered to all participants to assess the severity of depressive and anxiety symptoms.

### 2.3 Cerebral Image Acquisition

Brain PET imaging was performed using a Siemens/CTI ECAT HR+ PET scanner. A bolus intravenous injection of [^18^F]UCB‐H was administered at a dose of 157.06 ± 8.96 MBq (equivalent to 0.26 ± 0.21 MBq/μg) [19]. A thermoplastic mask was used to minimize head movement during acquisition. Dynamic PET data were acquired in frames of increasing duration: 6 × 10 s, 8 × 30 s, 5 × 120 s, and 17 × 300 s, for a total acquisition time of 100 min. All PET images were reconstructed using filtered backprojection with a Hann filter (4.9 mm full width at half maximum [FWHM]), incorporating corrections for attenuation, dead time, random events, and scatter using standard software (ECAT 7.1, Siemens/CTI, Knoxville, TN). All participants underwent T1‐weighted MRI on a 3.0 T MAGNETOM Skyra scanner (Siemens, Germany).

### 2.4 Image Processing

T1‐weighted images were processed using the SPM12 PET‐PVC correction toolbox to generate quantified MRI and segmented images, which were then normalized to standard montreal neurological institute (MNI) space using unified segmentation. PET dynamic images were co‐registered with each participant's T1‐weighted structural image. Partial volume effect (PVE) correction was performed using the MG method within the PET‐PVC correction toolbox, with gray matter, white matter, cerebrospinal fluid, and other regions serving as regions of interest (ROI) masks. The modulated gray matter images were resampled to an isotropic voxel size of 2 mm to match the PET images and smoothed using an 8 × 8 × 8 mm Gaussian kernel.

### 2.5 Animals

Nine adult female mice in each group (7 months old) were included in this study, and all underwent measurement and analysis. They were group‐housed in individually ventilated cages under controlled conditions (22°C ± 1°C; 55% ± 5% humidity), on a 12 h light/dark cycle, with food and water ad libitum. Chronic restraint stress (CRS) mice were restrained for 4 h/day for 21 days in perforated 50‐mL centrifuge tubes with ventilation holes and a tail‐opening. During restraint, CRS mice were deprived of food and water; controls had ad libitum access.

Forced swimming test (FST): Mice swam for 6 min. Immobility was recorded during the last 4 min. Immobility was defined as floating with only nostrils exposed and minimal limb movement [20].

Open‐Field test (OFT): Mice were placed in a gray plastic arena (55 × 35 × 24.5 cm). Center entries, center time, and center distance were recorded over 10 min [21].

### 2.6 Animal Image Acquisition

Mice were anesthetized with isoflurane in a 3:7 O_2_/air mixture (5% induction; 1.5%–2.0% maintenance). List‐mode PET data were acquired on a Focus 220 micro‐PET scanner (CTI‐Siemens, Germany; 1.4 mm center‐of‐field resolution) for 60 min immediately after intravenous injection of [^18^F]UCB‐H (10.1 ± 0.5 MBq, 125 μL). A 10‐min transmission scan with a ^57^Co source was then performed for attenuation correction. After scanning, the catheter was removed and mice were returned to their home cages.

### 2.7 Statistical Analysis

All data were first tested for normality using the Shapiro–Wilk test. *p* > 0.05 indicated normality. Intergroup comparisons for continuous variables were conducted using two‐sample *t*‐tests for normally distributed data and Mann–Whitney *U* tests for non‐normally distributed data. All statistical analysis was performed using SPSS (version 25.0). *p* < 0.05 was considered significant.

Image data processing was performed using MATLAB (version 2022b). SUVR regional comparisons across 116 Automated Anatomical Labeling (AAL) regions were performed using two‐sample *t*‐tests. Given the exploratory nature of this pilot study and the limited sample size, we report nominal *p*‐values without correction for multiple comparisons, and all significant findings are interpreted as hypothesis‐generating rather than confirmatory. Post hoc power analysis was performed using GPower version 3.1.9.7 with *α* = 0.05 (two‐tailed), based on the observed effect sizes and sample sizes. Pearson's correlation analysis was performed to examine the relationship between SUVR values in the cerebrum and cerebellum. Total SUVR for seven brain networks described by Yeo et al. was calculated [22]. For voxel‐wise whole‐brain analysis, parameters were estimated separately for the cerebrum (gray matter mask) and cerebellum (cerebellar mask). Voxel‐wise intergroup differences and associations with clinical severity were assessed using two‐sample *t*‐tests and multiple regression models in SPM12. Statistical significance was determined using Gaussian random‐field correction (*p* < 0.05) [23]. FC and network analysis were performed using the GRETNA toolbox, with intergroup comparisons assessed via permutation testing.

## 3 Results

### 3.1 Neuropsychological Assessment

No significant differences in age or education years were found between the FMD and HC groups (Table 1). The mean HAMD score was significantly higher in the FMD group (22.86 ± 3.19) than in the HC group (2.56 ± 1.13; *p* < 0.001). The mean HAMA score was also significantly higher in the FMD group (17.29 ± 4.92) than in controls (2.00 ± 0.71; *p* < 0.001). These results confirm clinically significant depressive and anxiety symptoms in the FMD group.

**TABLE 1:** Baseline data of patients and healthy controls.

Project	FMD group	HC group	*p*
Total, *n*	7	9	
Age, years, mean (SD)	32.429 (7.678)	32.111 (6.509)	0.930^a^
Education, years, mean (SD)	16 (1)	16.667 (1.323)	0.287^a^
HAMD, mean (SD)	22.857 (3.185)	2.556 (1.130)	0.000^a^
HAMA, mean (SD)	17.286 (4.923)	2 (0.707)	0.000^a^

Abbreviations: FMD, first‐episode major depressive disorder; HAMA, Hamilton Anxiety Scale; HAMD, Hamilton Depression Scale; HC, healthy control.

^a^ Two‐sample *t*‐test.

### 3.2 SUVR Analysis

**FIGURE 1:**
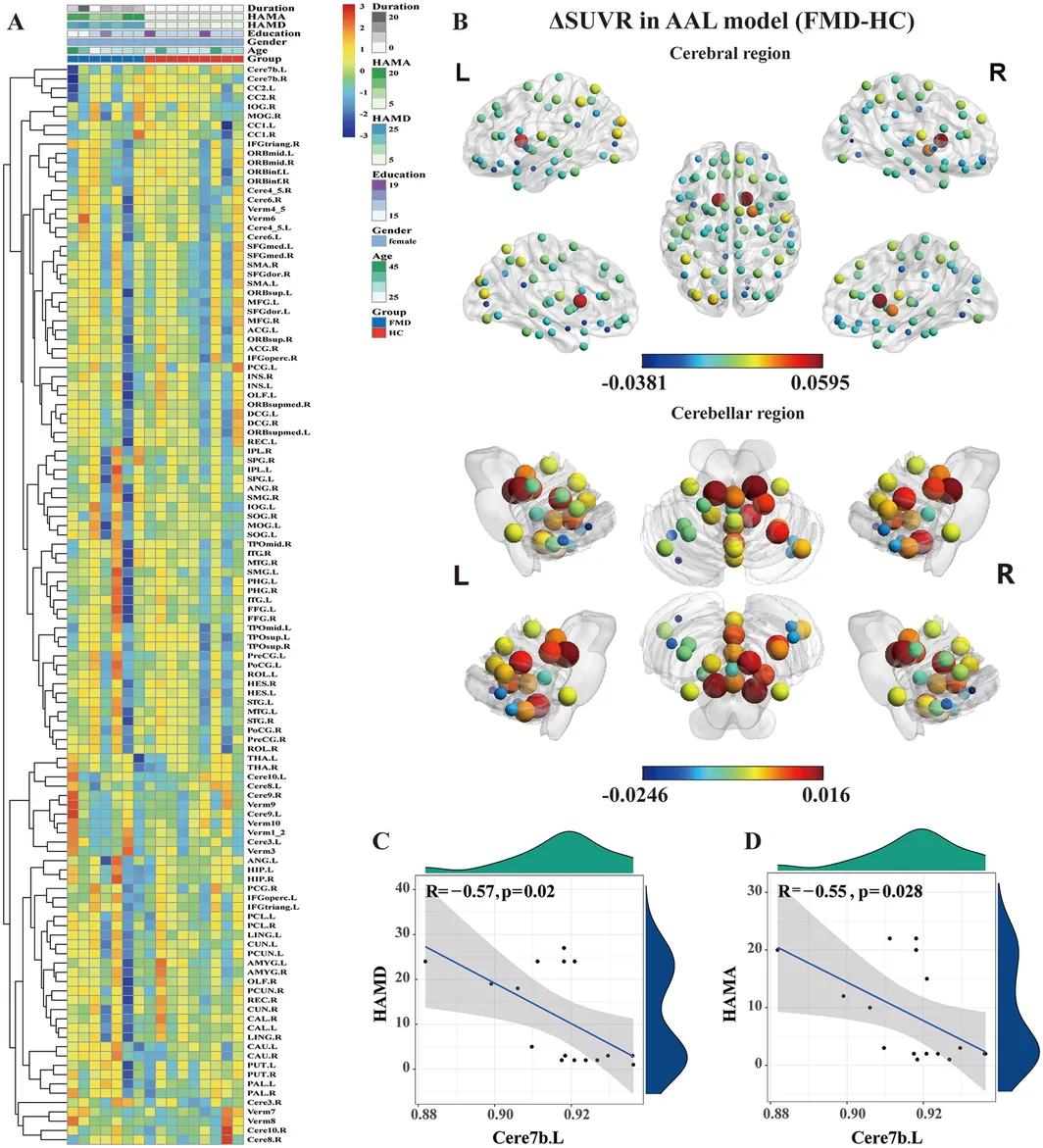
Intergroup differences in SUVR and correlation with clinical scores. (A) Heatmaps showing SUVR across 116 brain regions (AAL atlas) in FMD and HC groups. (B) Intergroup differences in SUVR (FMD—HC). The upper panel shows the cerebral regions, and the lower panel shows the cerebellar regions. (C, D) Correlations between SUVR in the Cere7b.L and clinical scores. (C) HAMD, (D) HAMA. AAL, automated anatomical labeling; FMD, first‐episode major depressive disorder; HAMA, Hamilton anxiety scale; HAMD, Hamilton depression scale; HC, healthy control; SUVR, standardized uptake value ratios.

SUVR values were calculated for 116 brain regions from the AAL atlas (Table S1). The FMD group showed nominally lower SUVR than the HC group in the left cerebellum 7b (Cere7b.L) (0.908 ± 0.014 vs. 0.924 ± 0.009, *p* = 0.012, uncorrected, Cohen's *d* = 1.46, power = 0.769). Intergroup differences are shown in Figure 1A,B. Across all brain regions, the greatest increase in the FMD group was in the right caudate nucleus (CAU.R) (1.091 ± 0.070 vs. 1.032 ± 0.052, Cohen's *d* = 0.99, power = 0.448), but the greatest decrease was in the right calcarine fissure and surrounding cortex (CAL.R) (1.175 ± 0.068 vs. 1.213 ± 0.050, Cohen's *d* = 0.65, power = 0.225). Within the cerebellum, the greatest increase was in the right cerebellum 3 (Cere3.R) (0.843 ± 0.021 vs. 0.827 ± 0.019, Cohen's *d* = 0.79, power = 0.309), but the greatest decrease was in the left cerebellum Crus2 (CC2.L) (0.911 ± 0.033 vs. 0.935 ± 0.025, Cohen's *d* = 0.86, power = 0.356). Cere7b.L SUVR were significantly negatively correlated with HAMA (*r* = −0.55, *p* = 0.028) and HAMD (*r* = −0.57, *p* = 0.020) scores (Figure 1C,D). Given the limited sample size, we restricted our correlation analyses to total HAMD and HAMA scores. The abbreviations of all brain regions are listed in Table S7.

After mapping AAL regions to the networks described by Yeo et al., total SUVR was calculated for each network. The FMD group showed the largest reduction in the ventral attention network (10.484 ± 0.538 vs. 10.537 ± 0.412, Cohen's *d* = 0.11, power = 0.055). The largest increase was in the somatomotor network (13.945 ± 0.755 vs. 13.853 ± 0.555, Cohen's *d* = 0.13, power = 0.057). However, neither difference was statistically significant. Network‐level SUVR comparisons are shown in Table S2.

### 3.3 Voxel‐Wise Analysis of Tracer Uptake

**FIGURE 2:**
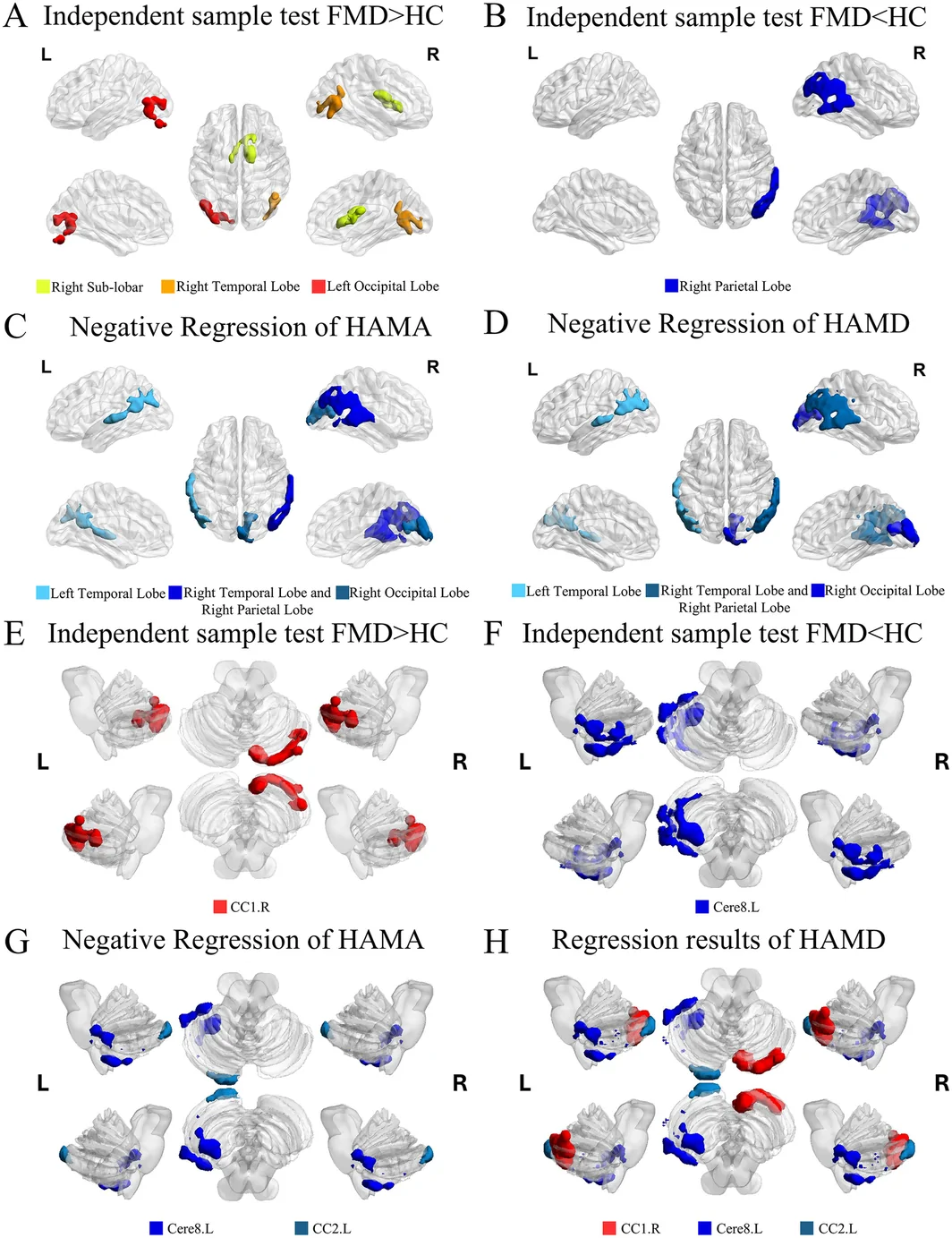
Differences in [^18^F]UCB‐H distribution volume between the FMD and HC groups and their association with clinical scores. (A) Brain regions showing significantly higher synaptic density in the FMD group compared to HCs. (B) Brain regions showing significantly lower synaptic density in the FMD group compared to HCs. (C, D) Brain regions showing a negative regression with HAMA (C) and HAMD (D) scores. (E) Cerebellar regions showing significantly higher synaptic density in the FMD group compared to HCs. (F) Cerebellar regions showing significantly lower synaptic density in the FMD group compared to HCs. (G) Cerebellar regions showing a negative regression with HAMA scores. (H) Cerebellar regions showing a significant regression with HAMD scores. The peak point locations of each cluster are marked in the panels. FMD, first‐episode major depressive disorder; HAMA, Hamilton anxiety scale; HAMD, Hamilton depression scale; HC, healthy control.

Voxel‐wise group comparisons revealed that the FMD group had significantly larger clusters than the HC group in the right Sub‐lobar, right temporal lobe, and left occipital lobe. In contrast, a significantly smaller cluster was found in the right occipital lobe (*p*_corrected < 0.05, cluster size > 563 voxels, Figure 2A,B). Within the cerebellum, the FMD group showed a significantly larger cluster in the right cerebellum Crus1 (CC1.R) and a significantly smaller cluster in the left cerebellum 8 (Cere8.L) (*p*_corrected < 0.05, cluster size > 173 voxels, Figure 2E,F).

Regression analysis revealed that the right temporal lobe, right parietal lobe, left temporal lobe, and right occipital lobe showed significant negative correlations with HAMA and HAMD scores (Figure 2C,D). No brain regions showed significant positive correlations with HAMA or HAMD scores. Within the cerebellum, significant negative correlations with HAMA and HAMD scores were found in Cere8.L and CC2.L. However, a significant positive correlation with HAMD scores was found in CC1.R. MNI coordinates, *k*‐values, and *t*‐values for these significant clusters are provided in Tables S3 and S4.

### 3.4 FC Analysis

**FIGURE 3:**
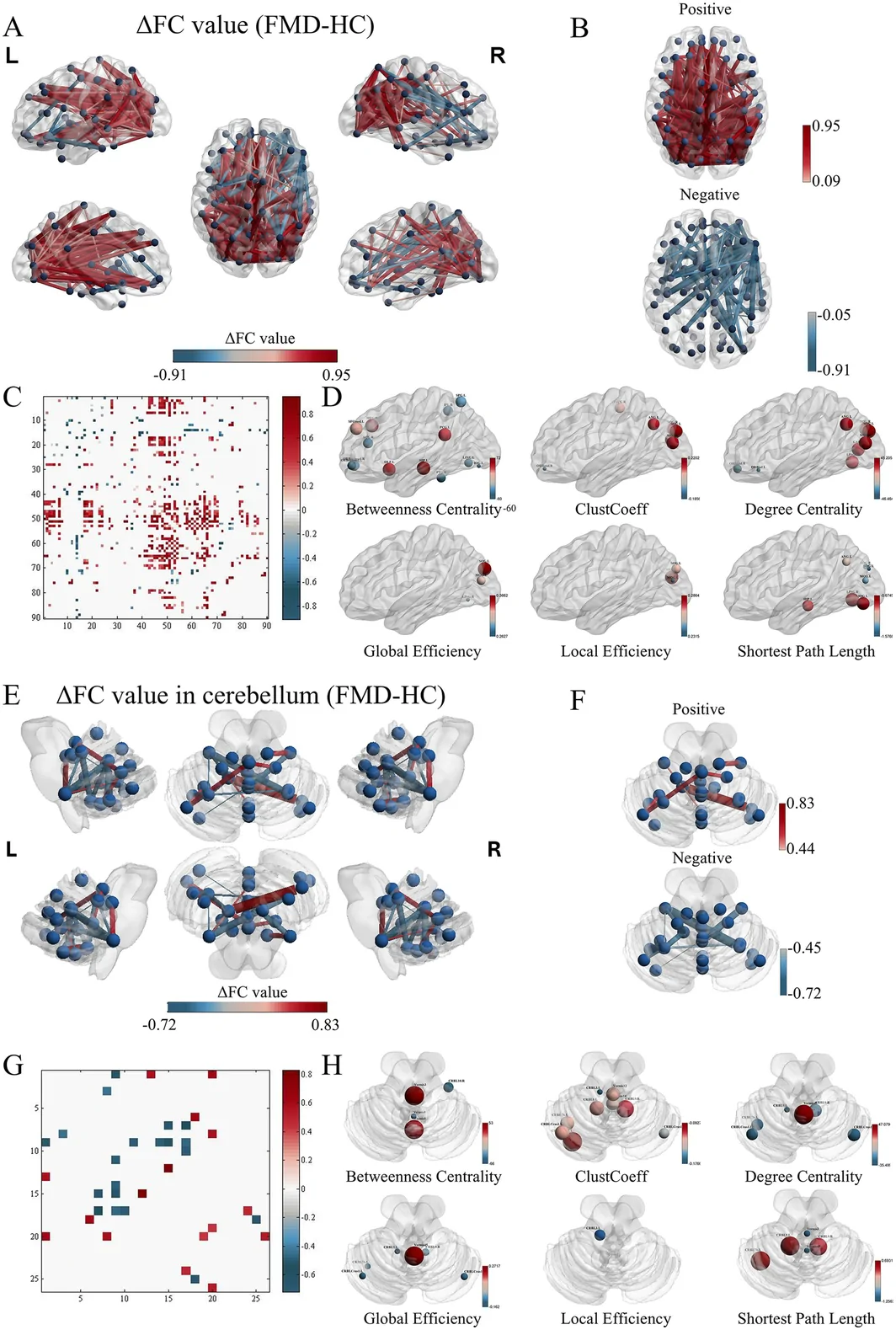
Analysis of FC differences. (A) Brain FC differences between the FMD and HC groups. (B) Decomposition of FC differences into positive and negative components. (C) Heatmap showing the matrix of FC differences. (D) Schematic representation of group differences in cerebral node topological properties, including betweenness centrality, clustcoeff, degree centrality, global efficiency, local efficiency, and shortest path length. (E) Cerebellar FC differences between the FMD and HC groups. (F) Decomposition of cerebellar FC differences into positive and negative components. (G) Heatmap showing the matrix of cerebellar FC differences. (H) Schematic representation of group differences in cerebellar node topological properties (same metrics as in D). FC, functional connectivity; FMD, first‐episode major depressive disorder; HC, healthy control.

FC changes within the cerebrum and cerebellum were calculated separately (Figure 3A). FCs that survived permutation testing were retained. A heatmap of these FC changes across 90 brain regions is shown in Figure 3C. To better highlight regions with increased or decreased connectivity in the FMD group, these differences were further decomposed into positive and negative components in Figure 3B. The FMD group showed significantly lower FC in the right prefrontal cortex and increased FC in middle and posterior brain regions. FC differences across 26 cerebellar regions are presented in Figure 3E–G. The cerebellar regions exhibiting altered connectivity were predominantly bilateral, underscoring the potential importance of interhemispheric coordination within the cerebellum. Group differences were also analyzed for six FC metrics. Significant regions for each metric are presented in Figure 3D (cerebrum) and 3H (cerebellum).

### 3.5 Network Analysis

**FIGURE 4:**
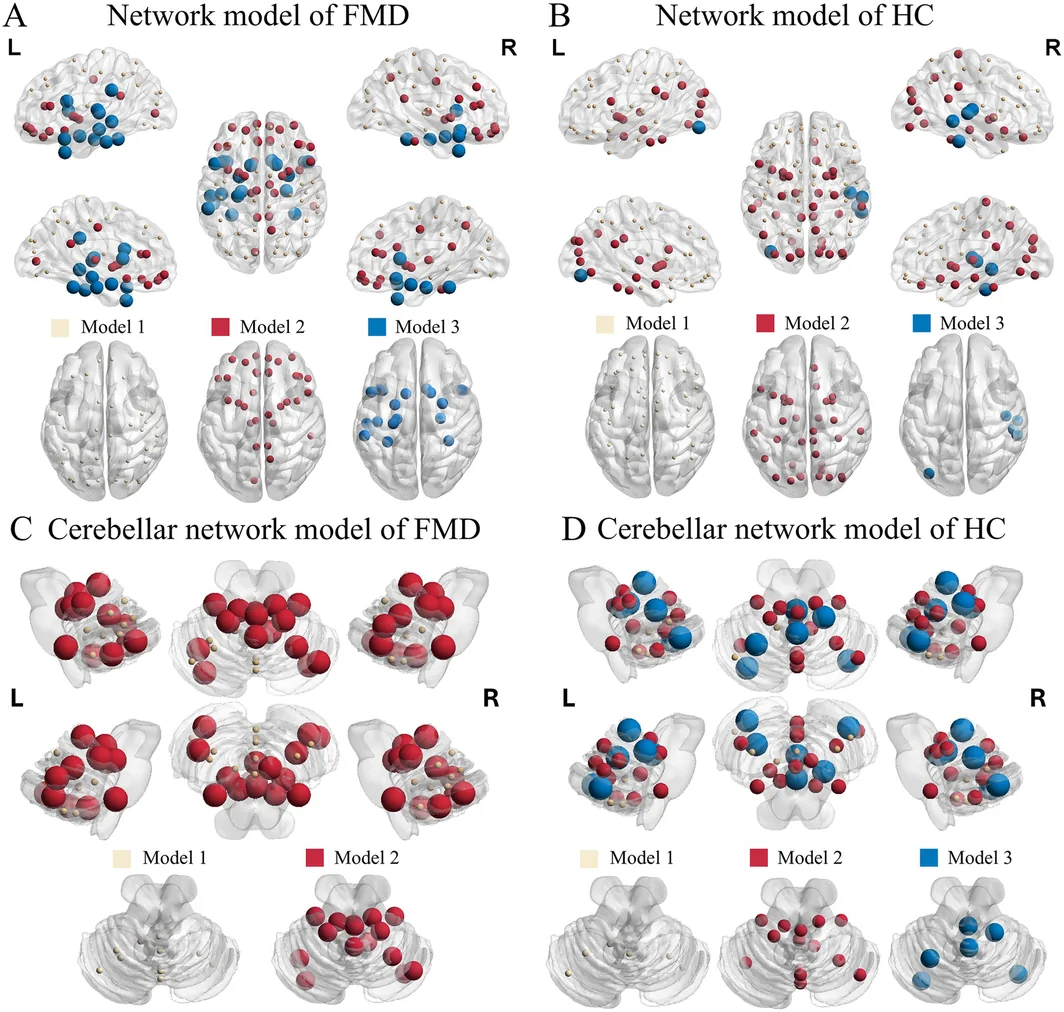
Schematic representation of resting‐state network modules. (A) Resting‐state network modules in the FMD group. (B) Resting‐state network modules in the HC group. (C) Cerebellar resting‐state network modules in the FMD group. (D) Cerebellar resting‐state network modules in the HC group. Separate networks are labeled in each panel. FMD, first‐episode major depressive disorder; HC, healthy control.

Three distinct brain network modules were identified in both the FMD and HC groups (Figure 4A,B). However, the FMD group concentrated on the bilateral prefrontal cortex within a single model. In the cerebellum, the cerebellar network in the FMD group showed both a reduced number of modules and a distinct organizational pattern compared with the HC group (Figure 4C,D). The brain and cerebellar regions included in each model across the two groups are listed in Tables S5 and S6.

### 3.6 Cerebello‐Cerebral Interaction Patterns in the FMD Group

**FIGURE 5:**
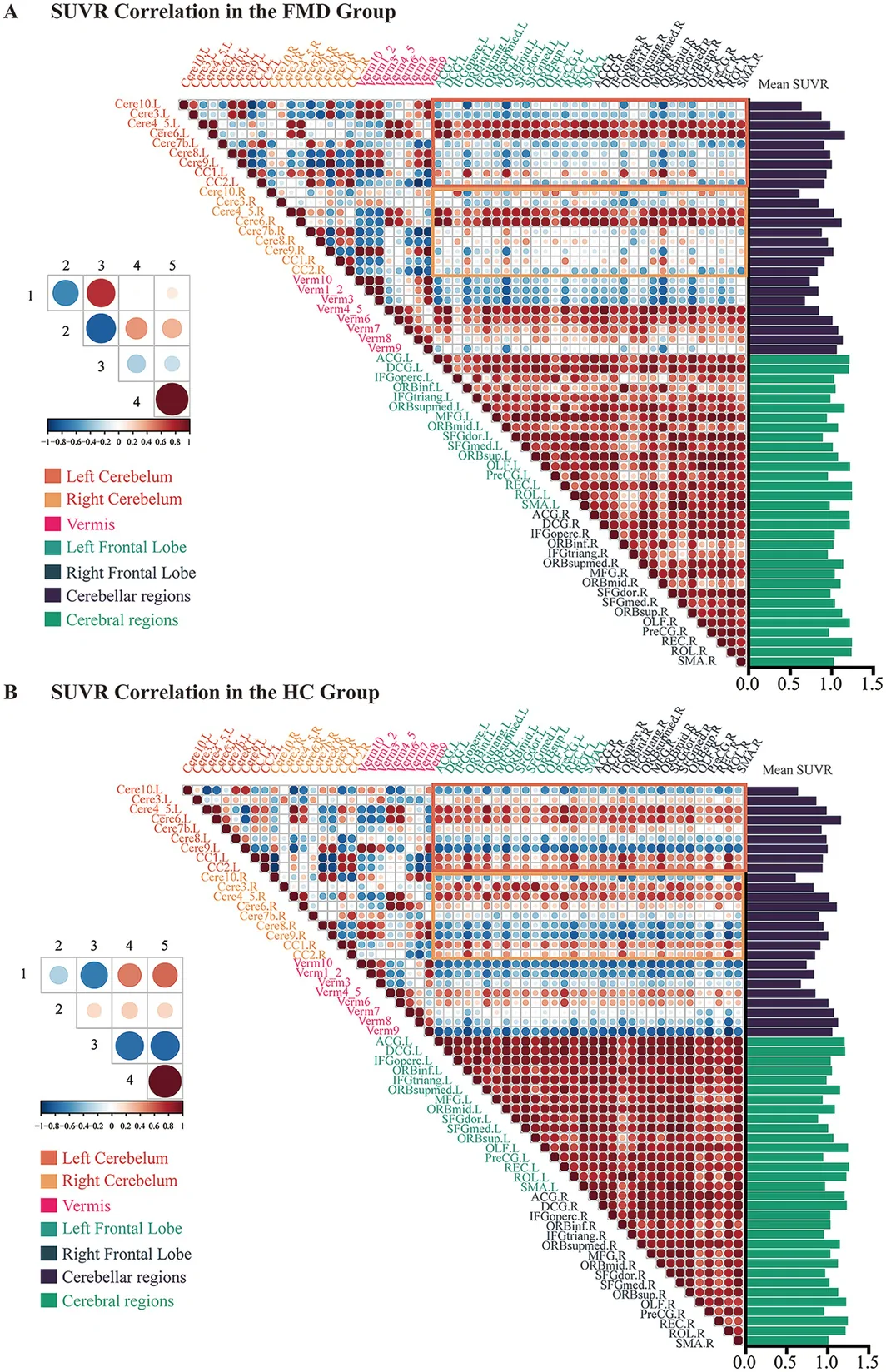
SUVR interaction map of the cerebellum and bilateral frontal lobes. (A) Heatmap showing SUVR correlations between bilateral frontal lobes and cerebellum in FMD group. (B) Heatmap showing SUVR correlations between bilateral frontal lobes and cerebellum in HC group. The orange box highlights the correlation between the left cerebellum and the bilateral frontal lobes. The yellow box highlights the correlation between the right cerebellum and the bilateral frontal lobes. FMD, first‐episode major depressive disorder; HC, healthy control; SUVR, standardized uptake value ratios.

Previous analyses examined SUVR, uptake differences, FC, and the brain network in the cerebrum and cerebellum separately. Building on this, we investigated cerebello‐cerebral interactions preliminarily. Given the importance of the frontal lobe in psychiatric disorders, we first analyzed the correlation between cerebellar SUVR and bilateral frontal lobe SUVR (Figure 5). The FMD group showed lower bilateral frontal lobe SUVR correlation than the HC group, particularly in left cerebellum Crus1 (CC1.L), CC2.L, left cerebellum 3 (Cere3.L), Cere7b.L, and CC1.R, right cerebellum Crus2 (CC2.R), Cere3.R, and right cerebellum 6 (Cere6.R). Conversely, vermis‐frontal lobe correlation was higher in the FMD group.

**FIGURE 6:**
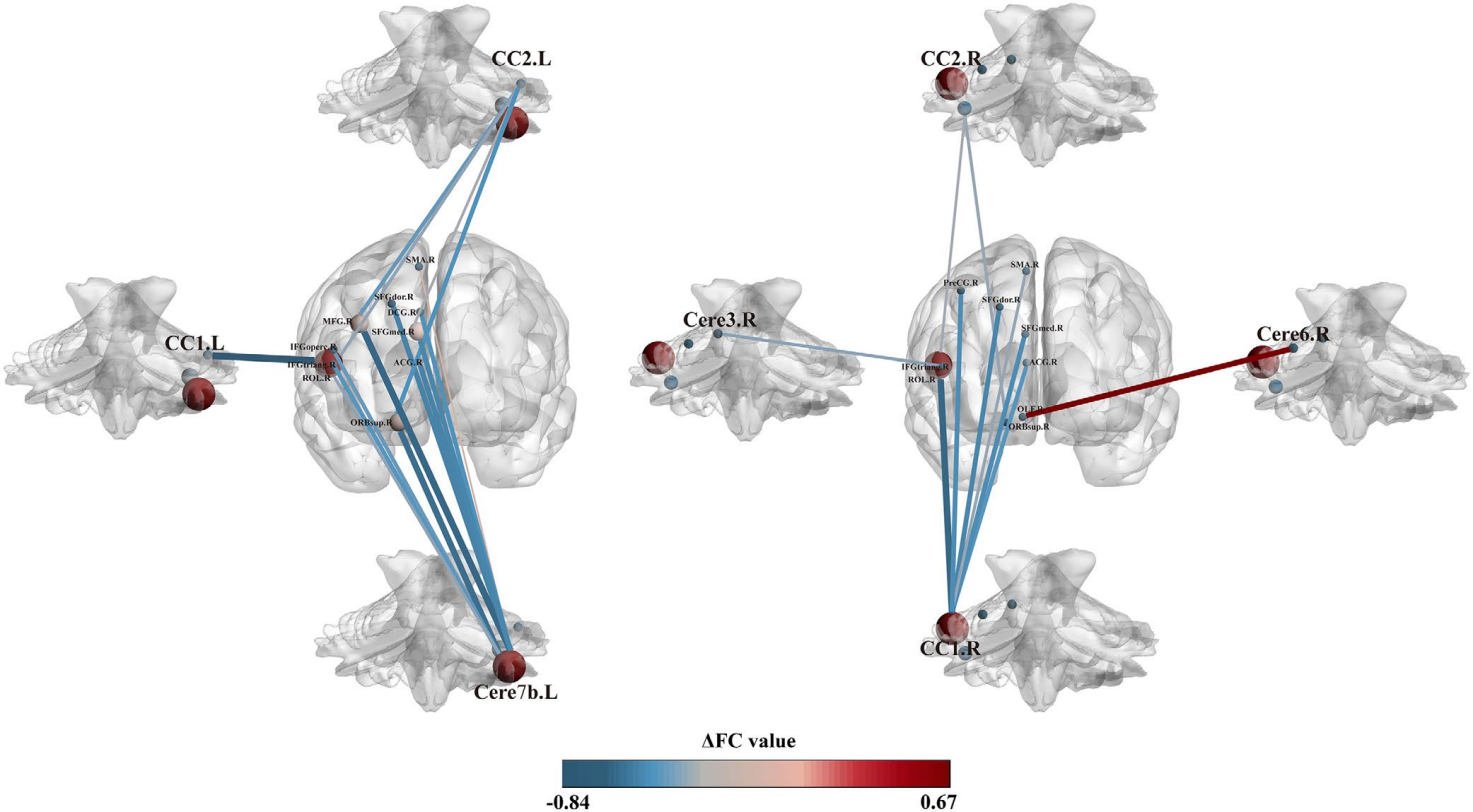
Functional connectivity changes between bilateral cerebellum and right frontal lobe. Left panel: Changes in left cerebellum‐right frontal lobe connectivity relative to healthy controls. Right panel: Changes in right cerebellum‐right frontal lobe connectivity relative to healthy controls. Node size reflects node degree (number of connections). Line color and thickness indicate the magnitude of connectivity changes.

To further investigate the relationship between the aforementioned cerebellar regions and the frontal lobe, we analyzed changes in cerebello‐frontal FC. Three categories of cerebellar regions were included: (1) cerebellar peak regions identified via between‐group difference analysis, (2) cerebellar peak regions identified via regression analysis, and (3) cerebellar regions showing significant changes in the SUVR correlation analysis described above. Because the significant decline in frontal lobe FC was predominantly confined to the right frontal lobe, subsequent analyses focused exclusively on this region. FC changes between each cerebellar region and the right frontal lobe, relative to the control group, are presented in Figure 6. The results indicate that CC1.L, CC2.L, and Cere7b.L all exhibited reduced FC with the right frontal lobe. Similarly, CC1.R, CC2.R, and Cere3.R showed a trend toward reduced FC with the right frontal lobe. In contrast, FC between Cere6.R and the right olfactory cortex (OLF.R) was increased. Overall, uncoupling between the left cerebellum and the right frontal lobe, manifested as decreased SUVR correlations and reduced FC, represents a pathological feature of MDD.

### 3.7 Metabolic Results of [ ^18^F]UCB‐H in CRS‐Induced Mouse Brain

Before human testing, we assessed the uptake of [^18^F]UCB‐H in a CRS mouse model to obtain preliminary findings and verify safety. After stress, the CRS group showed significantly longer FST immobility time and reduced OFT time and distance traveled in the center zone (Figure S1A–C). Representative locomotor trajectories are shown in Figure S1E. Subsequent PET imaging revealed significantly lower [^18^F]UCB‐H uptake in the brains of the CRS group. Representative PET images in the horizontal, coronal, and sagittal planes are shown in Figure S1D.

## 4 Discussion

This study investigated synaptic function in the cerebrum and cerebellum respectively of first‐episode, medication‐free female MDD patients. The study design was primarily based on established sex differences in the epidemiology, clinical presentation, and neurobiology of depression. Women are approximately twice as likely as men to develop depression, suggesting that sex hormones contribute to susceptibility [4] Moreover, depressed women exhibit more frequent atypical symptoms, stronger emotional responses, and more pronounced rumination, directly linked to the cerebellar‐cerebral circuits [24] By restricting the sample to women, we minimized confounding effects of sex‐related differences on synaptic and connectivity measures, which might otherwise be obscured in mixed‐sex analyses. Additionally, given the abundance of sex hormone receptors in the cerebellum and its sex‐specific developmental trajectories [25], the observed regional synaptic and connectivity changes may reflect female‐specific pathophysiological mechanisms. Nevertheless, these findings cannot be directly generalized to male patients, and future direct comparisons between male and female cohorts are warranted.

Decreased SUVR in the Cere7b.L negatively correlated with depression severity, suggesting that reduced cerebellar synaptic density may contribute to MDD. This finding aligns with the report by Sophie et al. [10] of reduced synaptic density in the prefrontal cortex, anterior cingulate, and hippocampus associated with depression severity, though cerebellar regions were not systematically examined. To our knowledge, since the first human [^18^F]UCB‐H study in 2015 [26], cerebellar synaptic dysfunction has been implicated in Alzheimer's disease [27], Parkinson's disease [28], and anxiety [29], but not in depression. Cere7b.L contains subregions involved in cognition, self‐referential processing, and emotional regulation. These subregions form functional circuits with brain regions supporting higher‐order cognition and emotional processing and are not directly connected to the somatic motor system [12]. Multiple neuroimaging modalities, including resting‐state functional magnetic resonance imaging, have linked structural and functional abnormalities in Cere7b.L to core depressive symptoms, such as cognitive impairment and emotional dysregulation [30, 31]. Among these, Tang et al. proposed that increased cerebellar neurite density in depressed patients with somatic symptoms may reflect adaptive changes [32]. Increased volume of the Cere7b.L has also been linked to improvement in depressive symptoms [30, 33]. However, neither regional volume nor neurite density directly reflects synaptic‐level alterations. Our findings offer preliminary imaging evidence at the synaptic level that reduced synaptic function in Cere7b.L may correlate with depression severity, though this result was obtained at an exploratory uncorrected threshold. Nevertheless, the negative correlation with clinical severity supports its potential relevance. The lower bilateral frontal lobe SUVR correlation and subsequent FC analysis further implicate this region at the network level. Independent replication in larger cohorts is required before definitive conclusions can be drawn.

Voxel‐based tracer uptake analysis, combined with multivariate regression of clinical symptoms, showed significantly reduced uptake in Cere8.L, negatively correlated with HAMD scores, and significantly increased uptake in CC1.R, positively correlated with HAMD scores. This bidirectional pattern in specific cerebellar regions may reflect subregional functional differentiation in depression pathophysiology. Özmen et al. reported reduced cortical thickness in cerebellar lobule VIII and increased thickness in Crus I in depressed patients [34], potentially providing a structural basis for the synaptic functional alterations observed here. Cere8.L, located in the primary sensorimotor area of the cerebellum, connects with cerebral cortical regions that govern traditional sensorimotor functions and basic emotional processing [35]. Li et al. reported that self‐sequencing activates Cere8.L more strongly than observing others [36]. Reduced synaptic function in Cere8.L may therefore reflect impaired self‐referential cognitive processing in depression. For instance, depressed patients with suicidal tendencies often exhibit blurred autobiographical memory, characterized by a lack of specificity when recalling past events [37]. In contrast to Cere8.L inactivation, CC1.R activity was significantly increased. The CC1.R connects with the default mode network (DMN) and frontoparietal control network, positioning it as a key node in higher‐order cognition and emotional regulation [35]. Treves et al. reported that enhanced connectivity between the cerebellum and the DMN in depression correlates with heightened rumination [38]. Enhanced synaptic function in CC1.R may reflect its excessive involvement in rumination. Interestingly, while synaptic function increased in CC1.R connected to the frontoparietal network, the synaptic function decreased in the right parietal lobe, a finding that correlated negatively with HAMD and HAMA scores. The frontoparietal control network governs attention regulation and goal‐directed behavior [39]. Decreased synaptic function in the right parietal lobe may explain attention deficits in MDD patients. Previous studies have demonstrated that neurofeedback training targeting the right parietal lobe can alleviate anxiety and depressive symptoms. In summary, synaptic loss in Cere8.L, a cognitive‐emotional cerebellar region, may drive self‐neglect and low mood, while enhanced synaptic function in CC1.R, a core DMN node, may underpin pathological rumination. These dual changes jointly define the cerebellar pathophysiological mechanism in women with MDD. Enhancing synaptic function in Cere8.L and the right parietal lobe, while therapeutically downregulating the pathologically elevated synaptic function in CC1.R, may represent a multimodal strategy for ameliorating depressive symptoms.

FC provides a framework for understanding how synaptic functional changes in distinct brain regions are coordinated. This study revealed significantly reduced FC between the right frontal lobe and multiple brain regions in depression. Further analysis demonstrated markedly weakened FC between the left middle frontal gyrus, orbital part (ORBmid.L) and the right hippocampus. This finding aligns with conclusions from a resting‐state functional magnetic resonance imaging study conducted by Lee et al. in adolescent patients with depression. That study also reported a reduction in FC between the right hippocampus and regions including the right insula and right middle frontal gyrus [40]. The interaction between the hippocampus and frontal cortex is increasingly recognized as a crucial link integrating cognitive and emotional processes [37]. At the cerebellar level, the most significant increase in FC was observed between the left cerebellum 9 (Cere9.L) and the right cerebellum 7b (Cere7b.R). Zhang et al. similarly reported elevated short‐range FC in cerebellar regions VII/IX among patients with depression [41]. However, whether this increased cerebellar connectivity serves as a compensatory mechanism for reduced cerebral connectivity remains inconclusive. Although Guo et al. suggest that increased cerebellar activity during rest in patients with MDD may represent a disease‐state phenomenon rather than a compensatory mechanism [42]. On the other hand, enhanced cerebellar FC following neuromodulation therapy correlates directly with clinical benefit, suggesting that the cerebellum may be recruited as an adaptive compensatory resource [43, 44]. Addressing this question will require real‐time neuroimaging techniques capable of simultaneously monitoring dynamic changes in cerebello‐cerebral FC, with particular attention to whether decreased cerebral connectivity and increased cerebellar connectivity occur synchronously. Network analysis, meanwhile, provides a framework for understanding how local FC gives rise to holistic patterns of brain organization. The present results indicate that cerebral network organization differs between patients with MDD and HCs, with the most prominent finding being a simplification of cerebellar networks in the patient group. Most studies of cerebellar networks have focused on their connectivity with cerebral networks, while paying less attention to the intrinsic organizational patterns of the cerebellar networks themselves [45, 46]. The simplification observed in cerebellar networks in the present study appears to be more indicative of pathological changes in MDD, and this simplified organization may underlie the reduced connectivity with cerebral networks. In depression, FC alterations are observed not only in affective circuits but also in sensorimotor, visual, auditory, and cerebellar networks [47].

After systematically comparing the distinguishing features of the cerebrum and cerebellum, we found that SUVR values in Cere7b.L were significantly reduced, accompanied by simplification of cerebellar network patterns, while FC in the right frontal lobe also showed a marked decrease. Based on these findings, we propose that abnormal interactions between the cerebellum and the right frontal lobe may represent a key pathological feature of MDD. To test this hypothesis, we first analyzed the correlation between cerebellar SUVR values and bilateral frontal lobe SUVR to identify cerebellar ROIs exhibiting interactive characteristics with the bilateral frontal lobes. Results showed that correlations were significantly reduced in the regions, including CC1.L, CC2.L, Cere3.L, Cere7b.L, CC1.R, CC2.R, Cere3.R and Cere6.R. Integrating these ROIs with the results of the previous differential uptake analysis, we also included Cere8.L as an ROI and further examined FC changes between the aforementioned cerebellar regions and the right frontal lobe. With the exception of Cere3.L and Cere8.L, all other cerebellar ROIs exhibited significant FC alterations with the right frontal lobe. It is worth noting that FC between the right cerebellum and the right frontal lobe exhibited bidirectional changes, with both increased and decreased connectivity observed simultaneously. In contrast, all ROIs in the left cerebellum showed a consistent FC decline, with the most significant decrease observed in the Cere7b.L. This lateralized difference may stem from the anatomical asymmetry of cerebellar‐cerebral projections. The classic cerebellar‐cerebral circuit follows a contralateral pattern [48], the left cerebellum primarily projects to the right cerebral hemisphere, while the right cerebellum exhibits broader ipsilateral projections [49], yielding more complex FC with the ipsilateral frontal lobe. Moreover, the degree of cerebellar functional asymmetry correlates significantly with cerebral asymmetry [50]. Given the previously observed marked FC reduction in the right frontal lobe, this may further exacerbate asymmetric functional dysregulation in the cerebellum. Therefore, we propose that uncoupling between the left cerebellum and the right frontal lobe, manifested specifically as reduced SUVR correlations and decreased FC, constitutes a key pathological feature of MDD. This finding is consistent with the results of a review by Malte et al. on MDD, which noted a significant FC weakening between cerebellar lobules VI and VIIA/B and the prefrontal cortex, and that this decline in cross‐regional connectivity is closely associated with impaired working memory and emotional regulation [12]. Furthermore, structural imaging studies by Chen et al. provide indirect support, indicating that as the depression progresses, gray matter atrophy may gradually extend from the ventromedial prefrontal cortex to the cerebellum, suggesting a dynamic association between lesions in these two regions [51]. Uncoupling between the left cerebellum and the right frontal lobe represents a core and reproducible neuroimaging marker of MDD, offering key insights into the neural mechanisms underlying the cognitive and emotional impairments associated with this disorder.

The present results should be interpreted with several limitations. First, only female participants were included, so generalizing findings to males warrants caution. Future studies should include both sexes to investigate potential gender differences in cerebello‐cerebral interactions. Second, recruiting first‐episode, medication‐free female MDD patients presented inherent challenges, resulting in a relatively small sample size that limits the ability to conduct subgroup analyses of clinical scales or to explore the relationship between symptom dimensions and imaging measures. Larger samples are needed in future studies. Third, while [^18^F]UCB‐H PET measures SV2A expression reflecting synaptic density, the observed SUVR and FC alterations cannot be attributed to specific excitatory versus inhibitory synaptic changes. Multimodal studies combining PET with electrophysiology or molecular markers are needed to elucidate the directionality of synaptic dysfunction. Fourth, uncorrected SUVR comparisons increase Type I error risk for Cere7b.L, and the post hoc power analysis falls slightly below 0.80, indicating Type II error risk. Further validation is required. At last, this animal study did not examine region‐specific effects or behavioral correlations, which merit future exploration.

## 5 Conclusion

This study employed [^18^F]UCB‐H PET imaging targeting SV2A to identify key brain regions with synaptic dysfunction in first‐episode female MDD patients. Using SUVR analysis, voxel‐based tracer uptake comparisons, FC analysis, and brain network analysis, we systematically characterized alterations in the cerebrum and cerebellum, respectively, of MDD patients and identified the contribution of synaptic changes to symptom severity. As cerebellar‐cortical interactions, uncoupling between the left cerebellum and the right frontal lobe represents a pathological hallmark of MDD. In summary, [^18^F]UCB‐H PET imaging effectively assesses in vivo synaptic alterations in the cerebello‐cerebral circuitry of MDD patients, highlighting the cerebellum's critical role in emotional processing.

## Author Contributions

**Juehan Wang:** data curation, formal analysis, writing of the original draft. **Yajun Lin:** data curation, methodology. **Tianfang Zhang:** conceptualization, validation. **Pengfeng Xu, Shanyan Lei:** data curation. **Hong Yang:** supervision, project administration. **Zuobing Chen:** supervision, project administration, and funding acquisition.

## Funding

This study was supported by grants from the National Key R&D Program of China (No. 2025YFC3608300; No. 2025YFC3608301), and the Zhejiang Key Laboratory of Intelligent Rehabilitation and Translational Neuroelectronics (Grant No. 2024E10108).

## Ethics Statement

This study protocol (approval no. IIT20230029C‐R1) was approved by the Ethics Committee of the First Affiliated Hospital of Zhejiang University School of Medicine. This animal experiment protocol (approval no. 2025‐1342) was approved by the Ethics Committee of the First Affiliated Hospital of Zhejiang University School of Medicine.

## Consent

Written informed consent was obtained from all participants in accordance with the Declaration of Helsinki.

## Conflicts of Interest

The authors declare no conflicts of interest.

## Supporting information


**Figure S1:** Depression‐like behavior and [^18^F]UCB‐H PET imaging results in the CRS mouse model. (A) immobility time in the forced swim test. (B) central zone residence time in the OFT. (C) Total distance traveled in the OFT. (D) Representative [^18^F]UCB‐H PET images in the horizontal, coronal, and sagittal planes. (E) Representative locomotor trajectories in the OFT. CRS, chronic restraint stress; OFT, open field test.
**Table S1:** The mean SUVR value across all 116 brain regions of the AAL atlas.
**Table S2:** The total SUVR values of each network between groups.
**Table S3:** The MNI coordinate position information of the gray mask density significantly differentiating clusters.
**Table S4:** The MNI coordinate position information of the cerebellum mask density significantly differentiating clusters.
**Table S5:** The brain regions in network.
**Table S6:** The cerebellar regions in network between groups.
**Table S7:** Abbreviations.

## Data Availability

Further inquiries of datasets and codes are available directed to the corresponding author.
