# Supplementary material for: Left Cerebellar‐Right Frontal Uncoupling in Female First‐Episode Major Depressive Disorder: Synaptic Evidence From [ 18F]UCB‐H PET
Source: CNS Neurosci Ther. 2026 Sep 4;32(9):e71144. doi: 10.1002/cns.71144 (PMC13545537; doi:10.1002/cns.71144)
Supplement: Supplementary file 1 [file CNS-32-e71144-s001.docx]

Supplementary Figure 1. Depression-like behavior and [18F]UCB-H PET imaging results in the CRS mouse model. (A) immobility time in the forced swim test. (B) central zone residence time in the OFT. (C) Total distance traveled in the OFT. (D) Representative [18F]UCB-H PET images in the horizontal, coronal, and sagittal planes. (E) Representative locomotor trajectories in the OFT. CRS, chronic restraint stress; OFT, open field test.

Supplementary Table 1 The mean SUVR value across all 116 brain regions of the AAL atlas

| ROIs | HC (avg±std) | FMD (avg±std) | P value | Cohen's d | Power |
| --- | --- | --- | --- | --- | --- |
| PreCG.L | 0.9439±0.04104 | 0.9532±0.05954 | 0.716^a^ | 0.18 | 0.063 |
| PreCG.R | 0.9569±0.04390 | 0.9658±0.05189 | 0.723^a^ | 0.19 | 0.064 |
| SFGdor.L | 0.8834±0.03935 | 0.8905±0.04281 | 0.736^a^ | 0.17 | 0.061 |
| SFGdor.R | 0.9685±0.04426 | 0.9831±0.04561 | 0.532^a^ | 0.33 | 0.094 |
| ORBsup.L | 1.0695±0.02930 | 1.0752±0.04319 | 0.536^b^ | 0.16 | 0.06 |
| ORBsup.R | 1.1262±0.04213 | 1.1245±0.05712 | 0.946^a^ | 0.03 | 0.05 |
| MFG.L | 0.9374±0.04427 | 0.9417±0.04291 | 0.851^a^ | 0.1 | 0.054 |
| MFG.R | 1.0307±0.05163 | 1.0346±0.04636 | 0.878^a^ | 0.08 | 0.053 |
| ORBmid.L | 1.0858±0.04885 | 1.0751±0.05449 | 0.692^a^ | 0.21 | 0.067 |
| ORBmid.R | 1.1239±0.06493 | 1.1067±0.07875 | 0.918^b^ | 0.24 | 0.073 |
| IFGoperc.L | 1.0352±0.03896 | 1.0296±0.06064 | 0.825^a^ | 0.11 | 0.055 |
| IFGoperc.R | 1.0297±0.04693 | 1.0338±0.04224 | 0.758^b^ | 0.09 | 0.053 |
| IFGtriang.L | 0.9856±0.03558 | 0.9832±0.04186 | 0.904^a^ | 0.06 | 0.051 |
| IFGtriang.R | 0.9514±0.03597 | 0.9527±0.05086 | 0.918^b^ | 0.03 | 0.05 |
| ORBinf.L | 1.0520±0.05174 | 1.0437±0.05307 | 0.759^a^ | 0.16 | 0.06 |
| ORBinf.R | 1.0328±0.05851 | 1.0221±0.06559 | 0.918^b^ | 0.17 | 0.061 |
| ROL.L | 1.2261±0.06025 | 1.2438±0.08657 | 0.918^b^ | 0.24 | 0.073 |
| ROL.R | 1.2159± 0.05963 | 1.2379±0.07507 | 0.524^a^ | 0.33 | 0.094 |
| SMA.L | 0.9667±0.05660 | 0.9744±0.05926 | 0.797^a^ | 0.13 | 0.057 |
| SMA.R | 1.0085±0.05602 | 1.0244± 0.05935 | 0.595^a^ | 0.28 | 0.081 |
| OLF.L | 1.2473±0.04903 | 1.2204±0.06572 | 0.362^a^ | 0.47 | 0.14 |
| OLF.R | 1.2239±0.04025 | 1.2182±0.06835 | 0.839^a^ | 0.11 | 0.055 |
| SFGmed.L | 1.0049±0.06540 | 1.0118±0.06687 | 0.839^a^ | 0.1 | 0.054 |
| SFGmed.R | 1.0191±0.06336 | 1.0378±0.06623 | 0.578^a^ | 0.29 | 0.084 |
| ORBsupmed.L | 1.1498±0.07552 | 1.1530±0.07415 | 0.933^a^ | 0.04 | 0.051 |
| ORBsupmed.R | 1.1471±0.06182 | 1.1369±0.07107 | 0.770^a^ | 0.15 | 0.059 |
| REC.L | 1.2618±0.06128 | 1.2409±0.07593 | 0.552^a^ | 0.31 | 0.089 |
| REC.R | 1.2460±0.05010 | 1.2427±0.07064 | 0.912^a^ | 0.06 | 0.051 |
| INS.L | 1.2162±0.05539 | 1.1991±0.07505 | 0.606^a^ | 0.26 | 0.077 |
| INS.R | 1.2570±0.06170 | 1.2272±0.08370 | 0.425^a^ | 0.41 | 0.118 |
| ACG.L | 1.2038±0.10105 | 1.2141±0.08987 | 0.836^a^ | 0.11 | 0.055 |
| ACG.R | 1.2018±0.07719 | 1.2129±0.07605 | 0.758^b^ | 0.14 | 0.058 |
| DCG.L | 1.2109±0.08665 | 1.2097±0.07494 | 0.978^a^ | 0.01 | 0.054 |
| DCG.R | 1.2327±0.08425 | 1.2183±0.07618 | 0.730^a^ | 0.18 | 0.063 |
| PCG.L | 1.1368±0.05575 | 1.1166±0.05325 | 0.476^a^ | 0.37 | 0.105 |
| PCG.R | 1.0246±0.02786 | 1.0215±0.03732 | 0.852^a^ | 0.1 | 0.054 |
| HIP.L | 1.0575±0.02999 | 1.0558±0.05760 | 0.939^a^ | 0.04 | 0.051 |
| HIP.R | 1.0574±0.03043 | 1.0604±0.06457 | 0.901^a^ | 0.06 | 0.051 |
| PHG.L | 1.0604±0.03372 | 1.0578±0.06233 | 0.915^a^ | 0.05 | 0.051 |
| PHG.R | 1.1336±0.04809 | 1.1152±0.08584 | 0.593^a^ | 0.27 | 0.079 |
| AMYG.L | 1.2421±0.07619 | 1.2170±0.08118 | 0.535^a^ | 0.32 | 0.091 |
| AMYG.R | 1.2121±0.06291 | 1.2094±0.07548 | 0.941^a^ | 0.04 | 0.051 |
| CAL.L | 1.1656±0.04401 | 1.1447±0.07452 | 0.494^a^ | 0.35 | 0.099 |
| CAL.R | 1.2127±0.04963 | 1.1746±0.06793 | 0.215^a^ | 0.65 | 0.225 |
| CUN.L | 1.1542±0.03971 | 1.1479±0.07610 | 0.833^a^ | 0.11 | 0.055 |
| CUN.R | 1.1709±0.03963 | 1.1560±0.07461 | 0.614^a^ | 0.26 | 0.077 |
| LING.L | 1.2186±0.03673 | 1.2166±0.08087 | 0.947^a^ | 0.03 | 0.05 |
| LING.R | 1.1355±0.03306 | 1.1171±0.07121 | 0.501^a^ | 0.35 | 0.099 |
| SOG.L | 1.0683±0.02453 | 1.0932±0.07429 | 0.358^a^ | 0.48 | 0.144 |
| SOG.R | 1.0859±0.01700 | 1.0892±0.06722 | 0.888^a^ | 0.07 | 0.052 |
| MOG.L | 1.1377±0.02507 | 1.1611±0.09089 | 0.469^a^ | 0.37 | 0.105 |
| MOG.R | 1.0949±0.02726 | 1.0959±0.07613 | 0.972^a^ | 0.02 | 0.05 |
| IOG.L | 1.1680±0.03774 | 1.1831±0.07282 | 0.597^a^ | 0.27 | 0.079 |
| IOG.R | 1.1245±0.04823 | 1.1358±0.07555 | 0.719^a^ | 0.18 | 0.063 |
| FFG.L | 1.2735±0.03769 | 1.2836±0.09920 | 0.783^a^ | 0.14 | 0.058 |
| FFG.R | 1.2589±0.05263 | 1.2616±0.10677 | 0.947^a^ | 0.03 | 0.05 |
| PoCG.L | 0.9610±0.03725 | 0.9729±0.06609 | 0.655^a^ | 0.23 | 0.071 |
| PoCG.R | 0.9726±0.03964 | 0.9796±0.06378 | 0.792^a^ | 0.14 | 0.058 |
| SPG.L | 0.9444±0.04013 | 0.9632±0.08985 | 0.408^b^ | 0.28 | 0.081 |
| SPG.R | 0.8443±0.03227 | 0.8561±0.07813 | 0.687^a^ | 0.21 | 0.067 |
| IPL.L | 1.0991±0.04076 | 1.1221±0.09324 | 0.515^a^ | 0.34 | 0.096 |
| IPL.R | 1.0794±0.03299 | 1.0952±0.09043 | 0.634^a^ | 0.25 | 0.075 |
| SMG.L | 1.1443± 0.04084 | 1.1525±0.07699 | 0.787^a^ | 0.14 | 0.058 |
| SMG.R | 1.1726±0.02743 | 1.1653±0.08101 | 0.803^a^ | 0.13 | 0.057 |
| ANG.L | 1.1430±0.04036 | 1.1330±0.10231 | 0.791^a^ | 0.14 | 0.058 |
| ANG.R | 1.1006±0.02050 | 1.0911±0.07441 | 0.717^a^ | 0.19 | 0.064 |
| PCUN.L | 1.0794±0.04743 | 1.0855±0.07557 | 0.846^a^ | 0.1 | 0.054 |
| PCUN.R | 1.0756±0.03777 | 1.0756±0.06392 | 0.837^b^ | 0 | 0.05 |
| PCL.L | 0.8523±0.04383 | 0.8539±0.05257 | 0.946^a^ | 0.03 | 0.05 |
| PCL.R | 0.8781±0.04688 | 0.8798±0.05721 | 0.946^a^ | 0.03 | 0.05 |
| CAU.L | 1.0025±0.05658 | 1.0541±0.10010 | 0.212^a^ | 0.66 | 0.231 |
| CAU.R | 1.0317±0.05171 | 1.0912±0.07009 | 0.070^a^ | 0.99 | 0.448 |
| PUT.L | 1.3018±0.05775 | 1.3076±0.08578 | 0.873^a^ | 0.08 | 0.053 |
| PUT.R | 1.3044±0.05606 | 1.3073±0.08555 | 0.935^a^ | 0.04 | 0.051 |
| PAL.L | 1.1794±0.04895 | 1.1867±0.07524 | 0.817^a^ | 0.12 | 0.056 |
| PAL.R | 1.1946±0.05184 | 1.2313±0.06838 | 0.241^a^ | 0.62 | 0.209 |
| THA.L | 1.0099±0.04284 | 1.0093±0.06794 | 0.983^a^ | 0.01 | 0.05 |
| THA.R | 1.0123±0.04090 | 1.0220±0.05547 | 0.693^a^ | 0.2 | 0.066 |
| HES.L | 1.2589±0.07447 | 1.2640±0.08130 | 0.918^b^ | 0.07 | 0.052 |
| HES.R | 1.2521±0.05005 | 1.2459±0.06984 | 0.837^a^ | 0.1 | 0.054 |
| STG.L | 1.1749±0.06861 | 1.1851±0.08036 | 0.788^a^ | 0.14 | 0.058 |
| STG.R | 1.1513±0.04335 | 1.1383±0.06565 | 0.640^a^ | 0.24 | 0.073 |
| TPOsup.L | 0.9631±0.09701 | 0.9609±0.07743 | 0.837^b^ | 0.02 | 0.05 |
| TPOsup.R | 0.9523±0.07078 | 0.9572±0.07005 | 0.837^b^ | 0.07 | 0.052 |
| MTG.L | 1.1454±0.03905 | 1.1536±0.06564 | 0.760^a^ | 0.16 | 0.06 |
| MTG.R | 1.1841±0.03125 | 1.1928±0.07292 | 0.749^a^ | 0.16 | 0.06 |
| TPOmid.L | 1.0158±0.08106 | 1.0184±0.09526 | 0.918^b^ | 0.03 | 0.05 |
| TPOmid.R | 0.9543±0.04830 | 0.9615±0.08368 | 0.830^a^ | 0.11 | 0.055 |
| ITG.L | 1.2504± 0.03936 | 1.2500±0.08671 | 0.990^a^ | 0.01 | 0.05 |
| ITG.R | 1.2630±0.04214 | 1.2648±0.09918 | 0.962^a^ | 0.02 | 0.05 |
| CC1.L | 0.9391±0.02476 | 0.9365±0.01359 | 0.351^b^ | 0.13 | 0.057 |
| CC1.R | 0.9091±0.01946 | 0.9121±0.02195 | 0.777^a^ | 0.15 | 0.059 |
| CC2.L | 0.9354±0.02489 | 0.9108±0.03306 | 0.055^b^ | 0.86 | 0.356 |
| CC2.R | 0.8443±0.02665 | 0.8301±0.04225 | 0.427^a^ | 0.41 | 0.118 |
| Cere3.L | 0.8608±0.01501 | 0.8755±0.03266 | 0.248^a^ | 0.61 | 0.204 |
| Cere3.R | 0.8268±0.01945 | 0.8428±0.02110 | 0.145^a^ | 0.79 | 0.309 |
| Cere4_5.L | 0.9864±0.02587 | 0.9804±0.03047 | 0.676^a^ | 0.21 | 0.067 |
| Cere4_5.R | 1.0178±0.02999 | 1.0265±0.04346 | 0.644^a^ | 0.24 | 0.073 |
| Cere6.L | 1.1622±0.02137 | 1.1569±0.03221 | 0.700^a^ | 0.2 | 0.066 |
| Cere6.R | 1.1085±0.02160 | 1.1190±0.03083 | 0.351^b^ | 0.4 | 0.115 |
| Cere7b.L | 0.9243±0.00880 | 0.9079±0.01381 | 0.012^a*^ | 1.46 | 0.769 |
| Cere7b.R | 0.8917±0.02500 | 0.8788±0.05520 | 0.837^b^ | 0.32 | 0.091 |
| Cere8.L | 0.9853±0.00908 | 0.9797±0.01077 | 0.282^a^ | 0.57 | 0.184 |
| Cere8.R | 0.9482±0.01740 | 0.9529±0.01260 | 0.252^b^ | 0.3 | 0.086 |
| Cere9.L | 0.9982±0.02030 | 1.0000±0.04227 | 0.910^a^ | 0.06 | 0.051 |
| Cere9.R | 1.0065±0.02446 | 1.0191±0.03347 | 0.397^a^ | 0.44 | 0.129 |
| Cere10.L | 0.6362±0.01837 | 0.6364±0.02383 | 0.987^a^ | 0.01 | 0.05 |
| Cere10.R | 0.6099±0.02273 | 0.6096±0.01441 | 0.975^a^ | 0.02 | 0.05 |
| Verm1_2 | 0.8336±0.05592 | 0.8394±0.09688 | 0.758^b^ | 0.08 | 0.053 |
| Verm3 | 0.6704±0.01740 | 0.6763±0.03587 | 0.669^a^ | 0.22 | 0.069 |
| Verm4_5 | 0.8471±0.01646 | 0.8461±0.02341 | 0.924^a^ | 0.05 | 0.051 |
| Verm6 | 1.0082±0.01751 | 1.0089±0.02967 | 0.954^a^ | 0.03 | 0.05 |
| Verm7 | 1.0762±0.02003 | 1.0778±0.01661 | 0.862^a^ | 0.09 | 0.053 |
| Verm8 | 1.1242±0.01918 | 1.1321±0.01741 | 0.114^b^ | 0.43 | 0.125 |
| Verm9 | 1.0554±0.03434 | 1.0597±0.04596 | 0.835^a^ | 0.11 | 0.055 |
| Verm10 | 0.7414±0.11873 | 0.7333±0.15325 | 0.907^a^ | 0.06 | 0.051 |

ROI, region of interest; HC, health control; FMD, first-episode major depressive disorder; avg, average; std, standard deviation. ^a^ Two-sample t-test, ^b^ Mann-Whitney U-test, ^*^ P < 0.05.

Supplementary Table 2 The total SUVR values of each network between groups

| Networks | HC (avg±std) | FMD (avg±std) | P value | Cohen's d | Power |
| --- | --- | --- | --- | --- | --- |
| Visual Network | 23.027 ± 0.423 | 23.077 ± 1.200 | 0.918^a^ | 0.06 | 0.051 |
| Somatomotor Network | 13.853 ± 0.590 | 13.944 ± 0.817 | 0.799^a^ | 0.13 | 0.057 |
| Dorsal Attention Network | 1.790 ± 0.069 | 1.820 ± 0.166 | 0.666^a^ | 0.25 | 0.074 |
| Ventral Attention Network | 10.538±0.436 | 10.484±0.580 | 0.836^a^ | 0.11 | 0.055 |
| Limbic Network | 21.313±0.778 | 21.336±1.233 | 0.965^a^ | 0.02 | 0.050 |
| Frontoparietal Network | 9.323±0.366 | 9.346±0.424 | 0.911^a^ | 0.06 | 0.051 |
| Default Mode Network | 19.552±0.790 | 19.577±1.014 | 0.957^a^ | 0.03 | 0.050 |

HC, health control; FMD, first-episode major depressive disorder; avg, average; std, standard deviation. ^a^ Two-sample t-test, ^*^ P < 0.05.

Supplementary Table 3. The MNI coordinate position information of the gray mask density significantly differentiating clusters

| Type | Cluster | Region | MNI coordinate | | | K | T |
| --- | --- | --- | --- | --- | --- | --- | --- |
|  |  |  | x | y | z |  |  |
| Independent sample test FMD>HC | 1 | Right Sub-lobar | 12 | -2 | 22 | 591 | 3.58 |
|  | 2 | Right Temporal Lobe | 42 | -64 | 4 | 611 | 2.93 |
|  | 3 | Left Occipital Lobe | -28 | -80 | -20 | 643 | 2.71 |
| Independent sample test FMD<HC | 1 | Right Parietal Lobe | 42 | -78 | 34 | 1264 | 3.72 |
| Negative Regression of HAMA | 1 | Right Temporal Lobe and  Right Parietal Lobe | 42 | -76 | 32 | 1790 | 5.11 |
|  | 2 | Left Temporal Lobe | -68 | -26 | 6 | 638 | 4.25 |
|  | 3 | Right Occipital Lobe | 4 | -90 | 12 | 940 | 3.45 |
| Negative Regression of HAMD | 1 | Right Temporal Lobe and  Right Parietal Lobe | 42 | -76 | 32 | 1963 | 4.81 |
|  | 2 | Left Temporal Lobe | -56 | -72 | 34 | 639 | 3.63 |
|  | 3 | Right Occipital Lobe | 6 | -90 | 12 | 961 | 3.47 |

MNI, Montreal Neurological Institute; HC, health control; FMD, first-episode major depressive disorder; HAMD, Hamilton Depression Scale; HAMA. Hamilton Anxiety Scale.

| Type | Cluster | Region | MNI coordinate | | | K | T |
| --- | --- | --- | --- | --- | --- | --- | --- |
|  |  |  | x | y | z |  |  |
| Independent sample test FMD>HC | 1 | CC1.R | 18 | -82 | -30 | 221 | 3.49 |
| Independent sample test FMD<HC | 1 | Cere8.L | -32 | -48 | -58 | 872 | 5.16 |
| Negative Regression of HAMA | 1 | Cere8.L | -30 | -48 | -58 | 789 | 4.96 |
|  | 2 | CC2.L | -10 | -86 | -30 | 164 | 3.42 |
| Positive Regression of HAMD | 1 | CC1.R | 6 | -76 | -32 | 183 | 3.47 |
| Negative Regression of HAMD | 1 | Cere8.L | -32 | -48 | -58 | 856 | 4.84 |
|  | 2 | CC.L | -10 | -86 | -30 | 205 | 3.6 |

Supplementary Table 4. The MNI coordinate position information of the cerebellum mask density significantly differentiating clusters

MNI, Montreal Neurological Institute; HC, health control; FMD, first-episode major depressive disorder; HAMD, Hamilton Depression Scale; HAMA. Hamilton Anxiety Scale.

Supplementary Table 5. The brain regions in network

| Models | FMD group | | HC group | |
| --- | --- | --- | --- | --- |
|  | Left hemisphere | Right hemisphere | Left hemisphere | Right hemisphere |
| Model 1 | SFGdor.L  MFG.L  ROL.L  SMA.L  SFGmed.L  CUN.L  LING.L  SOG.L  MOG.L  IOG.L  PoCG.L  SPG.L  IPL.L  ANG.L  PCUN.L  PCL.L | PreCG.R  SFGdor.R  ROL.R  SMA.R  SFGmed.R  HIP.R  CAL.R  CUN.R  LING.R  MOG.R  SOG.R  PoCG.R  SPG.R  IPL.R  SMG.R  IOG.R  ANG.R  PCL.R  HES.R  STG.R  MTG.R | PreCG.L  SFGdor.L  ORBsup.L  MFG.L  ORBmid.L  IFGtriang.L  ORBinf.L  ROL.L  SMA.L  OLF.L  SFGmed.L  ORBsupmed.L  REC.L  ACG.L  DCG.L  PHG.L  PoCG.L  SPG.L  IPL.L  PCUN.L  PCL.L  CAU.L  HES.L  STG.L  TPOsup.L  MTG.L  TPOmid.L | PreCG.R  SFGdor.R  ORBsup.R  MFG.R  ORBmid.R  IFGoperc.R  IFGtriang.R  ORBinf.R  ROL.R  SMA.R  SFGmed.R  ORBsupmed.R  INS.R  ACG.R  DCG.R  PHG.R  PoCG.R  SPG.R  IPL.R  ANG.R  CAU.R  TPOsup.R  TPOmid.R |
| Model 2 | ORBsup.L  ORBmid.L  IFGtriang.L  ORBinf.L  OLF.L  ORBsupmed.L  REC.L  INS.L  ACG.L  DCG.L  PCG.L  CAL.L  PUT.L  PAL.L  THA.L | ORBsup.R  MFG.R  ORBmid.R  IFGoperc.R  IFGtriang.R  ORBinf.R  ORBsupmed.R  REC.R  INS.R  ACG.R  DCG.R  PCG.R  PCUN.R  PUT.R  PAL.R  THA.R  ITG.R | IFGoperc.L  INS.L  PCG.L  HIP.L  AMYG.L  CAL.L  CUN.L  LING.L  SOG.L  MOG.L  FFG.L  SMG.L  ANG.L  PUT.L  PAL.L  THA.L  ITG.L | OLF.R  REC.R  PCG.R  HIP.R  AMYG.R  CAL.R  CUN.R  LING.R  SOG.R  MOG.R  IOG.R  FFG.R  SMG.R  PCUN.R  PCL.R  PUT.R  PAL.R  THA.R |
| Model 3 | IFGoperc.L  HIP.L  PHG.L  AMYG.L  FFG.L  SMG.L  CAU.L  HES.L  STG.L  TPOsup.L  MTG.L  TPOmid.L  ITG.L | OLF.R  PHG.R  AMYG.R  FFG.R  CAU.R  TPOsup.R  TPOmid.R | IOG.L | HES.R  STG.R  MTG.R  ITG.R |

Supplementary Table 6. The cerebellar regions in network between groups

| Models | FMD group | | | HC group | | |
| --- | --- | --- | --- | --- | --- | --- |
|  | Left hemisphere | Vermis | Right hemisphere | Left hemisphere | Vermis | Right hemisphere |
| Model 1 | CC1.L  Cere6.L  Cere8.L  Cere9.L | Verm6  Verm7  Verm8  Verm9  Verm10 | Cere7b.R  Cere8.R | CC1.L  Cere8.L  Cere9.L | Verm9 | Cere7b.R  Cere8.R |
| Model 2 | CC2.L  Cere3.L  Cere4_5.L  Cere7b.L  Cere10.L | Verm1_2  Verm3  Verm4_5 | CC1.R  CC2.R  Cere4_5.R  Cere6.R  Cere3.R  Cere9.R  Cere10.R | Cere3.L  Cere4_5.L  Cere7b.L  Cere10.L | Verm3  Verm6  Verm7  Verm8  Verm10 | CC1.R  Cere3.R  Cere6.R  Cere9.R  Cere10.R |
| Model 3 |  |  |  | CC2.L  Cere6.L | Verm1_2  Verm4_5 | CC2.R  Cere4_5.R |

Supplementary Table 7 Abbreviations

| Full title | Abbreviations |
| --- | --- |
| Major depressive disorder | MDD |
| Positron emission tomography | PET |
| Chronic restraint stress | CRS |
| Standardized uptake value ratios | SUVR |
| Functional connectivity | FC |
| Hamilton depression scale | HAMD |
| Hamilton anxiety scale | HAMA |
| Synaptic vesicle glycoprotein 2A | SV2A |
| Healthy controls | HCs |
| International Classification of Diseases, 10th Revision | ICD-10 |
| Diagnostic and Statistical Manual of Mental Disorders, Fifth Edition | DSM-5 |
| Partial volume effect | PVE |
| Regions of interest | ROI |
| Forced swimming test | FST |
| Open‑Field test | OFT |
| Automated anatomical labeling | AAL |
| Default mode network | DMN |
| Left precental gyrus | PreCG.L |
| Right precental gyrus | PreCG.R |
| Left superior frontal gyrus, dorsolateral | SFGdor.L |
| Right superior frontal gyrus, dorsolateral | SFGdor.R |
| Left superior frontal gyrus, orbital part | ORBsup.L |
| Right superior frontal gyrus, orbital part | ORBsup.R |
| Left middle frontal gyrus | MFG.L |
| Right middle frontal gyrus | MFG.R |
| Left middle frontal gyrus, orbital part | ORBmid.L |
| Right middle frontal gyrus, orbital part | ORBmid.R |
| Left inferior frontal gyrus, opercular part | IFGoperc.L |
| Right inferior frontal gyrus, opercular part | IFGoperc.R |
| Left inferior frontal gyrus, triangular part | IFGtriang.L |
| Right inferior frontal gyrus, triangular part | IFGtriang.R |
| Left inferior frontal gyrus, orbital part | ORBinf.L |
| Right inferior frontal gyrus, orbital part | ORBinf.R |
| Left rolandic operculum | ROL.L |
| Right rolandic operculum | ROL.R |
| Left supplementary motor area | SMA.L |
| Right supplementary motor area | SMA.R |
| Left olfactory cortex | OLF.L |
| Right olfactory cortex | OLF.R |
| Left superior frontal gyrus, medial | SFGmed.L |
| Right superior frontal gyrus, medial | SFGmed.R |
| Left superior frontal gyrus, medial orbital | ORBsupmed.L |
| Right superior frontal gyrus, medial orbital | ORBsupmed.R |
| Left gyrus rectus | REC.L |
| Right gyrus rectus | REC.R |
| Left insula | INS.L |
| Right insula | INS.R |
| Left anterior cingulate and paracingulate gyri | ACG.L |
| Right anterior cingulate and paracingulate gyri | ACG.R |
| Left median cingulate and paracingulate gyri | DCG.L |
| Right median cingulate and paracingulate gyri | DCG.R |
| Left posterior cingulate gyrus | PCG.L |
| Right posterior cingulate gyrus | PCG.R |
| Left hippocampus | HIP.L |
| Right hippocampus | HIP.R |
| Left parahippocampal gyrus | PHG.L |
| Right parahippocampal gyrus | PHG.R |
| Left amygdala | AMYG.L |
| Right amygdala | AMYG.R |
| Left calcarine fissure and surrounding cortex | CAL.L |
| Right calcarine fissure and surrounding cortex | CAL.R |
| Left cuneus | CUN.L |
| Right cuneus | CUN.R |
| Left lingual gyrus | LING.L |
| Right lingual gyrus | LING.R |
| Left superior occipital gyrus | SOG.L |
| Right superior occipital gyrus | SOG.R |
| Left middle occipital gyrus | MOG.L |
| Right middle occipital gyrus | MOG.R |
| Left inferior occipital gyrus | IOG.L |
| Right inferior occipital gyrus | IOG.R |
| Left fusiform gyrus | FFG.L |
| Right fusiform gyrus | FFG.R |
| Left postcentral gyrus | PoCG.L |
| Right postcentral gyrus | PoCG.R |
| Left superior parietal gyrus | SPG.L |
| Right superior parietal gyrus | SPG.R |
| Left inferior parietal, but supramarginal and angular gyri | IPL.L |
| Right inferior parietal, but supramarginal and angular gyri | IPL.R |
| Left supramarginal gyrus | SMG.L |
| Right supramarginal gyrus | SMG.R |
| Left angular gyrus | ANG.L |
| Right angular gyrus | ANG.R |
| Left precuneus | PCUN.L |
| Right precuneus | PCUN.R |
| Left paracentral lobule | PCL.L |
| Right paracentral lobule | PCL.R |
| Left caudate nucleus | CAU.L |
| Right caudate nucleus | CAU.R |
| Left lenticular nucleus | PUT.L |
| Right lenticular nucleus | PUT.R |
| Left lenticular nucleus, pallidum | PAL.L |
| Right lenticular nucleus, pallidum | PAL.R |
| Left thalamus | THA.L |
| Right thalamus | THA.R |
| Left heschl gyrus | HES.L |
| Right heschl gyrus | HES.R |
| Left superior temporal gyrus | STG.L |
| Right superior temporal gyrus | STG.R |
| Left temporal pole: superior temporal gyrus | TPOsup.L |
| Right temporal pole: superior temporal gyrus | TPOsup.R |
| Left middle temporal gyrus | MTG.L |
| Right middle temporal gyrus | MTG.R |
| Left temporal pole: middle temporal gyrus | TPOmid.L |
| Right temporal pole: middle temporal gyrus | TPOmid.R |
| Left inferior temporal gyrus | ITG.L |
| Right inferior temporal gyrus | ITG.R |
| Left cerebellum Crus1 | CC1.L |
| Right cerebellum Crus1 | CC1.R |
| Left cerebellum Crus2 | CC2.L |
| Right cerebellum Crus2 | CC2.R |
| Left cerebellum 3 | Cere3.L |
| Right cerebellum 3 | Cere3.R |
| Left cerebellum 4_5 | Cere4_5.L |
| Right cerebellum 4_5 | Cere4_5.R |
| Left cerebellum 6 | Cere6.L |
| Right cerebellum 6 | Cere6.R |
| Left cerebellum 7b | Cere7b.L |
| Right cerebellum 7b | Cere7b.R |
| Left cerebellum 8 | Cere8.L |
| Right cerebellum 8 | Cere8.R |
| Left cerebellum 9 | Cere9.L |
| Right cerebellum 9 | Cere9.R |
| Left cerebellum 10 | Cere10.L |
| Right cerebellum 10 | Cere10.R |
| Vermis 1_2 | Verm1_2 |
| Vermis 3 | Verm3 |
| Vermis 4_5 | Verm4_5 |
| Vermis 6 | Verm6 |
| Vermis 7 | Verm7 |
| Vermis 8 | Verm8 |
| Vermis 9 | Verm9 |
| Vermis 10 | Verm10 |
